# Health Equity and Ethical Considerations in Using Artificial Intelligence in Public Health and Medicine

**DOI:** 10.5888/pcd21.240245

**Published:** 2024-08-22

**Authors:** Irene Dankwa-Mullan

**Affiliations:** 1 Department of Health Policy and Management, Milken Institute School of Public Health, The George Washington University, Washington, District of Columbia

## Abstract

This commentary explores the critical roles of health equity and ethical considerations in the deployment of artificial intelligence (AI) in public health and medicine. As AI increasingly permeates these fields, it promises substantial benefits but also poses risks that could exacerbate existing disparities and ethical challenges. This commentary delves into the current integration of AI technologies, underscores the importance of ethical social responsibility, and discusses the implications for practice and policy. Recommendations are provided to ensure AI advancements are leveraged responsibly, promoting equitable health outcomes and adhering to rigorous ethical standards across all populations.

**Summary**

**What is already known on this topic?**

Artificial intelligence (AI) is increasingly used in health care for diagnostics, predictive analytics, and personalized medicine, but it can exacerbate health disparities and ethical concerns if not carefully managed.

**What is added by this report?**

This commentary highlights the multifaceted approach and strategies to promote health equity and ethical use of AI, emphasizing community engagement, inclusive data practices, and transparent algorithms.

**What are the implications for public health practice?**

Implementing these strategies can ensure that AI benefits all populations equitably, enhancing trust and effectiveness in public health interventions and medical care.

## Introduction

The integration of artificial intelligence (AI) in public health and medicine is revolutionizing how health care and public health professionals approach health care delivery, disease prediction, population health, and patient care management (1). As these technologies evolve, they offer unprecedented opportunities for expanding precision health, enhancing efficiency, and optimizing effectiveness in health services (2). However, this integration also prompts critical discussions of the ethical use of AI and the imperative to ensure health equity. This commentary explores how AI is reshaping public health and medicine, concerns about bias, ethical challenges, and the importance of incorporating an equity lens in its deployment.

AI’s potential to transform health is immense, from improving diagnostic accuracy to personalizing treatment plans and predicting disease trends (2). Yet, as we stand on the brink of this technological revolution, it is crucial to address the ethical implications and ensure that these advancements benefit all sections of society equitably. The misuse or unethical application of AI can lead to increased disparities and further exacerbate adverse outcomes for socially and economically disadvantaged populations.

This commentary not only discusses the current applications and benefits of AI but also emphasizes the critical need to maintain a balance between innovation and ethical responsibilities. The commentary explores the historical context of technological transitions in health, examines the effect of AI on health equity, and provides actionable insights and recommendations to guide practitioners, policymakers, researchers, and developers. The aim is to foster a health care environment that not only embraces technological advancements but also upholds the highest standards of equity and ethical practice.

## Background on AI in Public Health and Medicine

### Historical perspective

The integration of technology in health care is not a novel concept. AI was initially described in the 1950s as expert computer systems that could mimic human intelligence (2). These systems were followed in the 2000s by the emergence of computer vision and machine learning (2). Even though researchers continued to explore AI technologies with the evolution of data, the rapid advancement and adoption of AI has come to represent a transformative shift in the landscape. Technological innovations such as the electronic health record (EHR) and medical imaging revolutionized medical diagnostics and patient record management (2). Today, AI builds on these foundational advancements by offering more sophisticated tools for data analysis and clinical decision-making.

### Current trends

AI is now being used across various facets of public health and medicine, substantially altering how health professionals engage with their patients, communities, and health data. Two key areas where AI is making a mark are diagnostic algorithms and predictive analytics (2). For example, AI algorithms are being increasingly used to diagnose diseases from imaging scans — with higher accuracy and speed than human radiologists (3). In predictive analytics, AI can forecast outbreaks of diseases (4), hospital readmission rates (5), and a patient’s risk of developing chronic illnesses (6) by analyzing vast datasets. In this era of precision medicine, AI can help in tailoring medical treatments to individual genetic profiles, potentially improving outcomes and minimizing side effects (7). Public health surveillance, disease forecasting, and epidemic modeling are increasingly becoming important areas for integration of AI-based tools (6). These applications showcase a few of AI’s potential to enhance the efficacy and precision of public health and clinical decision-making. However, they also bring to light the need for a robust framework to manage these technologies responsibly.

### Transition challenges

As the public health and health care sectors navigate their way through digital transformation, several challenges emerge. These include technology challenges, widening knowledge gaps, and overall hesitance and resistance to change. For example, integrating AI into any existing public health or health care infrastructure requires substantial technology upgrades, a robust data architecture, and staff training. Apart from providing upgrades, gaps in understanding AI technologies among health care providers can hinder their effective implementation. In addition to that, adapting to AI-driven methods requires changes in established workflows and practices, which often meet with resistance from traditional health care providers. As AI continues to evolve, the health care industry must not only keep pace with these technological changes but also anticipate future developments. Addressing these challenges head-on will be essential for leveraging AI to improve health outcomes while ensuring that such technologies are used ethically and equitably.

## The Importance of Promoting Health Equity and Addressing Bias in AI Applications

The potential of AI to transform public health and medicine is immense. Yet, as health professionals harness these technologies, they must also consider the implications on health equity and ethical practices. Health equity in the context of AI applications refers to the fair and just distribution of health technologies and their benefits (8). It ensures that all individuals have access to the same high-quality health care services, regardless of their socioeconomic status, race, sex or gender, ethnicity, disability status, or geographic location (8). The deployment of AI diagnostic tools for diabetic retinopathy primarily in well-resourced health care settings or among populations with insurance coverage exemplifies an unfair distribution of technology. This approach disproportionately benefits people with greater economic means and access while potentially excluding socially or economically disadvantaged populations that may have a higher prevalence of disease but lack the resources or insurance necessary to access such advanced diagnostic tools. Equity is the absence of systematic disparities in health, or in the social determinants of health, between groups with different levels of underlying social advantage such as wealth, power, privilege, and prestige (9). For AI to be truly transformative, it must not only advance health care and outcomes but do so in a way that bridges existing health disparities rather than widening them.

## Sources and Risk of Bias

One of the most noteworthy concerns with AI is the risk of bias in algorithms, which can inadvertently perpetuate existing health disparities. AI bias is a general concept that refers to the fact that an AI system has been designed in a way that makes the system’s decisions or use unfair (10). These AI data biases often arise from various sources, including the processes of data access, collection, acquisition, preparation, processing, development, and validation (11). Bias can also arise from the processes through which scientific evidence is generated, from lack of research diversity and from inadequate data governance. AI models are typically trained on available data, which may not adequately represent racial and ethnic minority groups or other populations that are medically underserved (11). For example, Obermeyer et al discovered that commercial algorithms, which use cost as a proxy for illness, exhibit racial bias by inadequately identifying the health needs of Black patients compared with White patients despite similar levels of chronic illnesses (12). Training data can also reflect historical biases in treatment and access to care for socially disadvantaged populations, leading AI to replicate these injustices (12). Finally, many AI tools are so-called black boxes — in which decision-making processes are not transparent — making it difficult to assess and rectify biases (13). These are some of the problems that underscore the need for meticulous oversight and corrective measures in the development and deployment of AI technologies to ensure they serve all populations equitably.

Even though addressing AI biases has primarily focused on algorithms, external sources of AI bias exist. They include experience and expertise, exclusion, environment, empathy, and evidence (14).

### Experience and expertise bias

Experience and expertise bias refers to the skew introduced by the varying levels of expertise among individuals involved in developing AI systems (14). This bias can manifest in several ways including:

- Training data quality: The quality of the training data can be influenced by the expertise of those who collect, label, and input the data. Inconsistent or incorrect labeling due to lack of expertise can lead to a biased model (2,11,15).
- Algorithm development: The design and tuning of algorithms require a high level of expertise. Inadequate expertise can result in models that do not generalize well across diverse populations (2).
- Clinical implementation: Varying levels of familiarity with AI tools among health care providers can affect how these tools are implemented and interpreted, potentially leading to biased outcomes (2,14,16).

### Exclusion bias

Exclusion bias occurs when certain groups are systematically left out of the data collection and analysis processes (14). This bias can result in AI systems that do not accurately represent or serve the entire population. Some examples are:

- Data missingness: When data are missing or incomplete for groups within a dataset, the AI system may not learn patterns relevant to these groups, leading to poorer performance for them compared with other groups (2,11).
- Underrepresentation: Exclusion of certain demographic groups in clinical trials or datasets can cause AI to be less effective or even harmful to these groups (2,11).
- Access to care: AI tools developed without considering socially or economically marginalized populations might not address the unique barriers these groups face in accessing health care (2,11).

### Environment bias

Environment bias arises from the socio-environmental context in which data are collected and used (14). This bias can include the following:

- Social determinants of health: Factors such as income, education, and living conditions can influence health outcomes and need to be adequately represented in datasets (2,11).
- Physical environment: Geographic and environmental factors (eg, urban vs rural settings) can affect health outcomes and must be considered to avoid biased AI predictions (2,11).
- Integration of environmental factors: Ensuring that environmental variables are incorporated into AI models can help in understanding and mitigating health disparities.

### Empathy bias

Empathy bias refers to the challenge of incorporating human experiences and subjective elements that are difficult to quantify into AI systems (14). This bias includes:

- Quantitative versus qualitative data: AI systems primarily rely on quantitative data, which can miss nuanced human experiences that affect health outcomes.
- Patient preferences: Empathy bias can occur when AI systems do not consider patient preferences, values, and unique circumstances, leading to recommendations that are misaligned with patient needs (14).
- Human stories: Integrating personal stories and experiences into AI models can enhance their relevance and fairness, although this factor presents a complex challenge.

### Evidence bias

Evidence bias involves the processes through which scientific evidence is generated, disseminated, and translated into practice (14). This bias can affect the overall reliability and applicability of AI systems. Examples include:

- Research funding: How research is funded can introduce biases, as funding priorities may not align with the needs of all populations.
- Publication bias: There is often a bias toward publishing positive results, which can skew the evidence base that AI systems rely on.
- Translation to practice: The way evidence is translated into clinical guidelines and policies can introduce biases if it does not consider the diversity of patient populations and contexts.

AI is not a monolithic entity; rather, it comprises various interconnected technologies and data inputs of intricate stacks playing a distinct role, contributing to the overall functionality, outputs, and intelligence of the system. To enhance clarity and understanding about sources of biases, it is beneficial to conceptualize the stack of interconnected technologies and inputs (Table). Biases that occur during the development of AI tools or models were mapped to specific points in the stack, to identify their origins and implement targeted strategies to address them (Table).

**Table:** Outline for Understanding Artificial Intelligence (AI) as a Stack of Interconnected Technologies and Where Biases Can Occur During the Development of AI Tools

Interconnected stack of AI technologies	Points where biases can occur	Reference
**Data and evidence generation**	• Experience and expertise bias • Exclusion bias • Environment bias • Empathy bias • Evidence bias	Dankwa-Mullan and Weeraratne (14)
**Model development**		
Data collection: gathering raw data from various sources (eg, sensors, user inputs, patient-reported outcomes, electronic health records and administrative claims databases, community health–related and social surveys, public health surveys, clinical trials, research data)	• Data sampling bias: Occurs when the data collected are not representative of the population of focus, leading to skewed insights. • Historical bias**:** Biases present in historical data can be perpetuated. For example, if past hiring practices favored certain demographic characteristics, a model trained on this data might continue to favor these characteristics.	Roski et al (2); Nazer et al (11)
Data preparation and preprocessing: cleaning, transforming, and structuring data for analysis	• Data cleaning bias: Bias can be introduced during the data cleaning process if certain data points are disproportionately removed or altered. For example, removing outliers might inadvertently exclude data on minority groups. • Feature selection bias: Occurs when choosing features that reflect existing prejudices or systemic biases. For example, using zip code as a feature in credit scoring might unintentionally introduce racial and/or socioeconomic bias.	Roski et al (2); Nazer et al (11)
Feature engineering: Creating relevant features from raw data to improve model performance	• Human bias in feature selection: The selection and creation of features can reflect the biases of the individuals involved in the process. For example, selecting features that favor certain groups over others: frequency of health care visits or access to specialists care can favor people with better access, and variables that measure engagement with digital health tools can favor younger or more tech-savvy populations. • Overfitting specific biases: Creating features that overfit the training data might capture and reinforce biases present in that data.	Chen et al (16)
Model selection: Choosing the appropriate algorithms and models for the task	• Algorithmic bias: Some algorithms might inherently favor certain patterns or demographic groups, which may lead to algorithmic bias. For example, decision trees might create splits that disproportionately affect certain demographics. • Inherent biases in model architecture: Certain model architectures may have biases based on their design. For example, linear models might fail to capture complex patterns in data related to underrepresented groups.	Roski et al (2); Nazer et al (11)
Model training: Training the model using prepared data	• Training data bias: Bias in the training data can lead to biased model outcomes. For example, if the training data contains biased labels, the model will learn and reproduce those biases. • Overfitting and underfitting: Overfitting to biased training data can exacerbate biases (by tailoring the model too closely to the training data), while underfitting might fail to capture important nuances, leading to a lack of fairness.	Roski et al (2); Yang et al (15)
Model evaluation and validation: Using metrics and validation techniques to assess the model’s performance	• Validation set bias: Bias in the evaluation process can arise if the validation set is not representative or if biased metrics are used to assess performance. In other words, if the validation set is not representative, it can lead to misleading performance metrics. For example, evaluating a model on a biased subset might indicate good performance while hiding biases. • Metric selection bias: This bias results from choosing evaluation metrics that do not capture fairness aspects. For example, using accuracy alone might ignore disparities in model performance across different groups.	Roski et al (2)
Model deployment: Integrating the trained model into production environments	• Deployment context bias: The deployment context can introduce bias if the model is used in a different environment than it was trained for, affecting its performance and fairness. The environment in which the model is deployed might differ from the training environment, introducing bias. For example, a model trained in one geographical area might not perform well in another. • Real-world feedback loop bias: As the model interacts with the real world, it might receive biased feedback, reinforcing existing biases. For example, a recommendation system might continue to favor popular items, ignoring niche interests.	Ferrara (17)
Monitoring and maintenance: Continuously monitoring model performance and making updates	• Drift in data distribution: As models are used over time, changes in data distributions can introduce new biases, and feedback loops can reinforce existing biases. Over time, the data distribution might change, leading to biases if the model is not updated. For example, shifts in consumer behavior can render an e-commerce model biased if it remains static. • Ongoing feedback bias: Continuous feedback loops can reinforce existing biases. For example, if a model’s recommendations are followed by users, the resulting data might further entrench those recommendations.	Roski et al (2); Ferrara (17)

To mitigate the risk of bias and promote health equity in AI, several strategic actions are recommended. These actions include collecting data from diverse population groups to ensure AI systems are well-informed and represent the variability in human health; developing AI with explainable outcomes to allow users to understand and trust decisions and ensure accountability in AI-driven processes; continuously monitoring AI systems for biased outcomes; and adjusting algorithms accordingly to ensure they remain equitable over time.

Specific proposed strategies for addressing bias follow.

### Addressing experience and expertise bias

- Diverse expert teams: Assemble multidisciplinary teams with diverse expertise, including data scientists, clinicians, ethicists, and social scientists, to inform, develop, and evaluate AI systems.
- Continuous training: Provide ongoing education and training for health care providers on AI technologies to ensure they are proficient in using and interpreting AI tools.
- Standardized protocols: Develop and adhere to standardized protocols for data collection, labeling, and algorithm development to minimize variability due to different levels of expertise.

### Addressing exclusion bias

- Inclusive data collection: Ensure datasets include diverse demographic groups by actively recruiting underrepresented populations in data collection efforts.
- Equity audits: Conduct regular equity audits of AI systems to identify and address any exclusion of populations.
- Accessible AI solutions: Design AI tools with accessibility in mind, ensuring that they cater to the needs of socially and economically marginalized populations and do not perpetuate existing barriers to care.

### Addressing environment bias

- Integration of social determinants: Include social determinants of health (eg, income, education, housing) in AI models to provide a more holistic understanding of health outcomes.
- Geospatial analysis: Use geospatial analysis to incorporate environmental factors such as air quality, water access, and neighborhood characteristics into health data.
- Contextual adaptation: Adapt AI models to local contexts, ensuring that they account for regional variations in social and environmental factors that affect health.

### Addressing empathy bias

- Incorporation of qualitative data: Combine quantitative data with qualitative insights from patient interviews, focus groups, and patient narratives to capture a full picture of health experiences.
- Patient-centered design: Engage patients in the design and development of AI systems to ensure that their preferences, values, and experiences are reflected in the models.
- Ethical review boards: Establish ethical review boards that include patient representatives to oversee the development and deployment of AI tools, ensuring they align with patient needs and ethical standards.

### Addressing evidence bias

- Diversification of funding: Advocate for diverse funding sources to support research that addresses the health needs of varied populations, avoiding biases introduced by funding priorities.
- Transparent reporting: Encourage transparent reporting of all research findings, including negative results, to build a comprehensive and unbiased evidence base.
- Inclusive guidelines: Develop clinical guidelines that are inclusive and consider the diverse patient populations and contexts in which they will be applied.

These strategies illustrate that while biases in the development and deployment of AI present challenges to health equity, with careful planning and ethical consideration AI also offers substantial opportunities to enhance health care for all. By prioritizing equity in the design and implementation of AI, public health professionals and medical practitioners can use these powerful tools to not only improve health outcomes but also ensure these improvements are shared across all segments of the population.

## Ethical Considerations in the Use of Artificial Intelligence

### Ethical frameworks

The deployment of AI in health necessitates adherence to established ethical frameworks designed to guide clinical practice and technological development (18,19). These frameworks typically emphasize principles that must be carefully considered when integrating AI into health care settings (18,19). Principles of beneficence and nonmaleficence ensure that AI technologies benefit patients and do not cause harm, whether through error, bias, or misuse (18,19). Another ethical AI principle is preserving patient autonomy by maintaining transparency and consent in AI interactions (18,19). Fairness and justice principles ensure that AI-driven tools do not create or exacerbate inequalities but rather promote equitable access to health care services (18,19).

### Privacy and confidentiality

With AI’s ability to process vast amounts of personal data, safeguarding patient privacy and confidentiality becomes paramount (18,19). These safeguards involve several key concerns about data security, informed consent, and misuse of data. It is critical to implement robust security measures to protect health data against unauthorized access and breaches (18,19). In addition, for populations with limited English proficiency, it is important to make sure informed consent forms are reviewed and explained to patients or translated. In this digital age, we can consider refining consent forms and including concise language for patients on how their data will be used in AI systems to inform their care. Finally, as part of ensuring privacy and confidentiality and limiting potential misuse, we should encourage collecting only data that are necessary for a specific AI application.

### Decision-making

AI’s role in clinical decision-making, public health interventions, and population health management introduces complexities in the extent of human oversight and the transparency of AI decisions. To ensure human oversight, we should establish guidelines for human oversight in AI-driven decisions, ensuring that machines augment rather than replace human judgment. To maintain trust and accountability, it is also important to develop AI systems whose actions can be understood and explained to practitioners and patients. Finally, determining how responsibilities and liabilities are shared among AI developers, health professionals, and institutions when AI is used in patient care is a complex and critical component of integrating AI into health care systems. This component involves understanding the roles and obligations of each partner to ensure patient safety, legal compliance, and ethical standards are upheld. Developers are responsible for creating accurate, reliable, and safe AI tools. Health care providers using AI tools must be adequately trained and responsible for interpreting AI outputs correctly, making final clinical decisions based on a combination of AI insights, patient values, and their professional judgment. Public health professionals must be guided by the principles of responsibility and ethics to enhance the ability to analyze data, predict health trends, and implement effective interventions to ensure the well-being of individuals and communities. Institutions need to establish policies and provide oversight to monitor AI performance, ensuring compliance with legal and ethical standards.

### Community engagement

Involving diverse communities in the AI development lifecycle is essential for its ethical application in public health and medicine. This approach ensures that AI systems are developed with a comprehensive understanding of the unique needs and challenges faced by various populations. Benefits of community engagement include enhanced relevance of the AI system to address the actual needs and preferences of the population, leading to better outcomes, and an increased trust and acceptance, with likelihood of successful implementation of the AI system (20).

## Challenges and Opportunities

The ethical integration of AI in health care and public health presents both challenges and opportunities. For example, AI can potentially streamline workflows and enhance diagnostic accuracy, but it also raises issues such as the potential for dehumanization in care and reduced patient–provider interactions (17). By addressing these ethical considerations proactively, working partners in public health and medicine can leverage AI to improve population health and health care outcomes while maintaining a commitment to ethical practice. As we delve into the transformative potential of AI in public health and medicine, it becomes increasingly apparent that while AI offers substantial benefits for health care efficiency and effectiveness, it also introduces substantial ethical and equity challenges.

To promote health equity and ethical AI use in public health and medicine, it is recommended to develop inclusive AI policies, enhance ethical frameworks, and ensure transparency and accountability (Figure). Investing in public and professional education about AI, fostering community engagement, and integrating social determinants of health into AI models are essential. Additionally, diverse funding for research and evidence, continuous monitoring and evaluation of AI systems, and interdisciplinary collaboration are crucial strategies to ensure AI technologies are fair, equitable, and beneficial for all populations (Figure).

**Figure:**
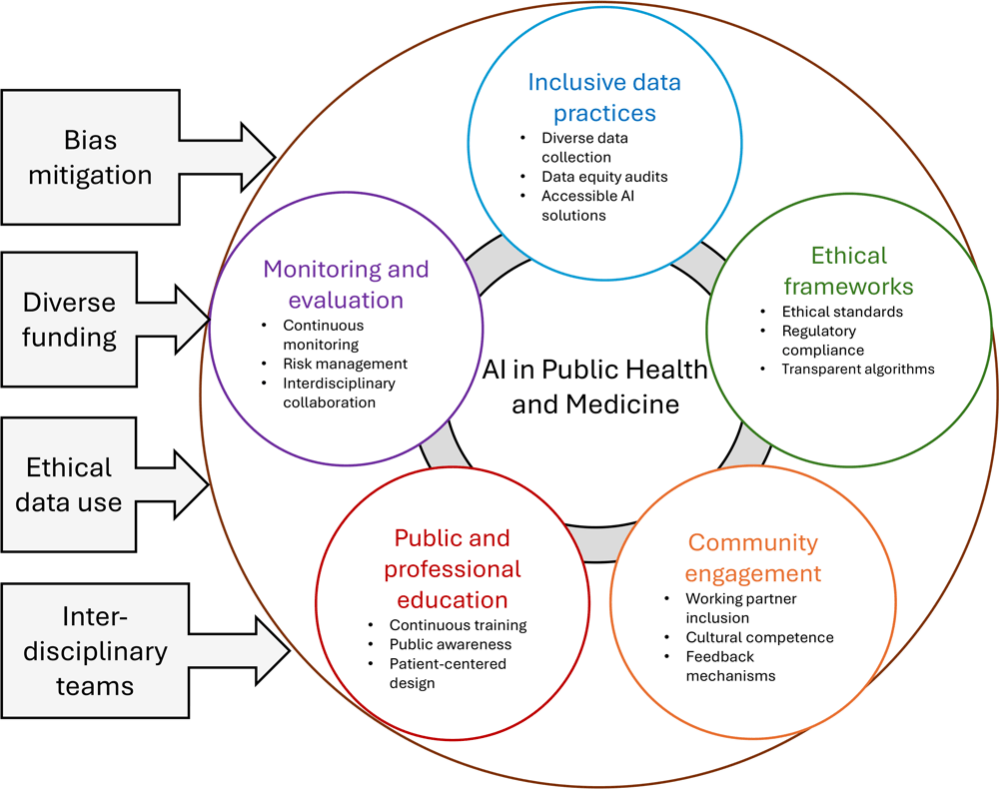
Multifaceted approach for ethical and equitable implementation of artificial intelligence (AI) in public health and medicine.

To advance public health and medicine responsibly, it is also imperative that partners work collaboratively to ensure that AI technologies not only meet the highest standards of innovation but also adhere to ethical and equitable practices. By implementing these recommendations, health care and public health professionals can leverage AI to enhance health care outcomes while safeguarding against potential inequalities and ethical transgressions.

This comprehensive approach ensures that AI serves as a tool for positive change, propelling public health and medicine into a future where technology and human values are aligned to promote the well-being of all individuals.
